# Amazon fig (*Ficus subapiculata*): A unconventional food plant with technological potential for the food industry

**DOI:** 10.1016/j.fochx.2025.102699

**Published:** 2025-06-30

**Authors:** Josias Martins dos Anjos Cruz, Renilto Frota Corrêa, Valdely Ferreira Kinupp, Michele Fernandes Pereira, Kidney de Oliveira Gomes Neves, Jojo Rodrigues, Bianca Muniz Lacerda Ventura, Tiago Antônio de Oliveira Mendes, Edgar Aparecido Sanches, Pedro Henrique Campelo, Jaqueline de Araújo Bezerra

**Affiliations:** a Graduate Program in Chemistry, Federal University of Amazonas (UFAM), Manaus, Amazonas, Brazil; b Center for Studies in Science and Technology in the Amazon (NECTAM), Manaus, Amazonas, Brazil; c Federal Institute of Education, Science and Technology of Amazonas (IFAM), Manaus, Amazonas, Brazil; d Soil and Plant Laboratory (LASP), Embrapa Amazônia Ocidental, Manaus, Amazonas, Brazil; e NMR Laboratory (NMRLab), Federal University of Amazonas, Manaus 69067-005, Brazil; f Department of Biochemistry and Molecular Biology, Federal University of Viçosa (UFV), Viçosa, Minas Gerais, Brazil; g Laboratory of Nanostructured Polymers (NANOPOL), Federal University of Amazonas, Manaus, Amazonas, Brazil; h Department of Food Technology, Federal University of Viçosa (UFV), Viçosa, Minas Gerais, Brazil

**Keywords:** Fruit, Composition, Mineral, Bioactive compounds, Antioxidant, Cytotoxicity

## Abstract

Amazon fig is an underexplored non-conventional food plant native to the Amazon region. The objective of this study was to determine its physicochemical properties, cytotoxicity, and chemical composition. The highest total phenolic content was observed in the hydroethanolic extract (709.5 mg GAE/g), which was associated with significant antioxidant potential against DPPH• (1539.1 μmol TE/g) and ABTS•^+^ (1994.9 μmol TE/g) radicals. No cytotoxic effects were observed in the tested cell lines. The fruit was primarily composed of carbohydrates and organic acids. Seven phenolic acids and four flavonoids were identified, including 4-hydroxybenzoic, protocatechuic and chlorogenic acids, quercetin and rutin. Among the mineral components, potassium (13.17 g/kg) and calcium (8.50 g/kg) were predominant. Volatile compound analysis identified 25 compounds, with β-elemene being the major constituent (8.37 %). Future research should focus on the quantification of bioactive compounds in this Amazonian matrix and the development of functional food products and ingredients for diverse applications.

## Highlights

• Amazon fig is a functional food and a source of flavonoids and phenolic acids.
• The fruits are important for meeting the body's needs for Mn, Cu, Ca, and Mg.
• Twenty-six volatile compounds were identified, mainly *β*-elemene.
• The fruit has potential for the development of functional food products.

## Graphical abstract

**Figure:**
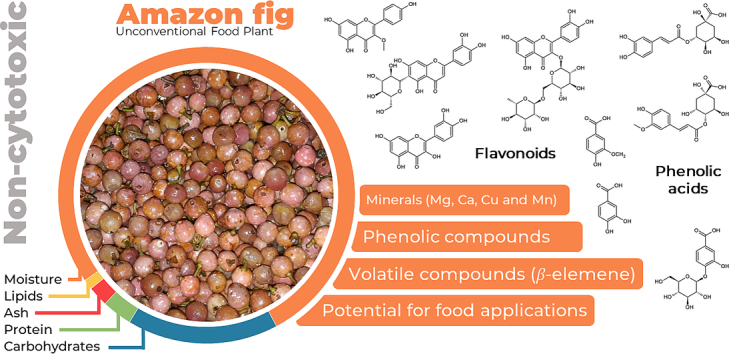

## 1 Introduction

The Amazon rainforest harbors an exceptional diversity of fruit-bearing botanical families, whose fruits exhibit a wide range of colors, flavors, and textures, reflecting their complex biochemical composition (Cruz et al., 2024). Among these families, Moraceae stands out, encompassing approximately 40 genera (Chaudhary et al., 2023), with *Ficus* being the largest, comprising over 1000 species. Commonly known as fig trees or “gameleiras” in Brazil (Santos & Ramalho, 1997), *Ficus* species display remarkable morphological diversity, ranging from small shrubs to large canopy trees distributed throughout tropical, subtropical, and temperate regions (Shi et al., 2018).

**Fig. 1:**
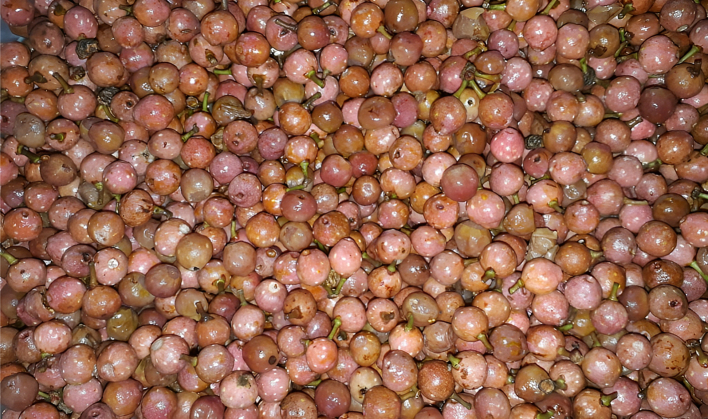
Fruits of *Ficus subapiculata* (Miq.) Miq.

These plants produce specialized inflorescence-derived structures known as figs, characterized by a soft, thin outer layer enclosing a fleshy interior that contains numerous flowers, which are pollinated by highly specialized wasps (Clement & Weiblen, 2009). Several fig species have been extensively studied, with reports highlighting the presence of anthocyanins, coumarins, flavonoids, organic acids, terpenoids, vitamins, fatty acids, sugars, and significant antioxidant activity (Cruz et al., 2022). However, other species remain poorly characterized, including *Ficus subapiculata* (Fig. 1).

*Ficus subapiculata* (Miq.) Miq. is a tree with a dense, globular canopy that produces tender, succulent, and mildly acidic infructescences, commonly referred to as minifig, Amazon fig, or “minifigo-da-campirana”. This species thrives in light, sandy soils of the Amazon region (Kinupp & Lorenzi, 2014). In a previous study, Amazon fig juice was subjected to thermal and ultrasonic treatments, which enhanced its *in vitro* antioxidant activity; however, its detailed chemical composition and cytotoxicity have yet to be elucidated (Cruz et al., 2023).

Therefore, this study aimed to characterize the chemical composition of Amazon fig (including mineral content, proximate composition, volatile compounds and other secondary metabolites), as well as its physicochemical properties, cytotoxicity, and antioxidant capacity. We hypothesized that Amazon fig holds technological potential as a functional ingredient for use in the food industry.

## 2 Materials and methods

### 2.1 Fruit collection and sample preparation

*Ficus subapiculata* fruits were collected at Sítio PANC, Manaus, Amazonas, Brazil, under SisGen authorization A92360E. Botanical identification was performed by Professor Valdely Ferreira Kinupp, Ph.D. Fresh, whole *Ficus subapiculata* fruits were randomly selected, weighed using an analytical balance, and measured with a caliper to determine their longitudinal dimensions.

A portion of the fresh fruits was reserved for physicochemical property analyses, volatile analysis and colorimetric assays. The remaining portion was lyophilized (Freeze dryer model SL-404, Solab) for 72 h at 233.15 K and − 93,325.7 Pa. The lyophilized material was then utilized for cytotoxicity assays, hydroethanolic extract preparation, and analysis by NMR and HRMS.

### 2.2 Physicochemical properties

The evaluated physicochemical parameters included moisture content by oven drying at 105 °C (Greenhouse model NT 513 D, Nova Técnica), ash content by muffle furnace incineration at 550 °C (Muffle furnace model SP-1200DRP7/G, SPLabor), protein content by the Kjeldahl method (Nitrogen distiller model SP-74, SPLabor), lipid content by Soxhlet extraction with hexane for 8 h (Sohxelt model Luca-145/6, Lucadema), and titratable acidity (Instituto Adolfo Lutz, 2008). Carbohydrate content was determined by difference (Ogunlaja et al., 2020), while color parameters were assessed following Castro et al. (2020) in DeltaVista spectrophotometer (DeltaColor). Additionally, soluble solids (Refractometer model HI96801, Hanna) and pH (Digital phmeter model AK90, Akso) were measured. Energy content was calculated using eq. 1 (Palmeira et al., 2019).

(1) $$\text{Energy}\;\left(\text{kcal}/100\;g\right)=4\times \left(\%\text{proteins}+\%\text{carbohydrates}\right)+9\times \left(\%\text{lipids}\right)$$

### 2.3 Mineral content

Atomic absorption spectroscopy was used to detect Ca, Mg, Fe, Mn, Zn and Cu, spectrometry for P, and flame photometry for K according to methodology adapted (Malavolta et al., 1989). The lyophilized samples (500 mg) were extracted by acid digestion with HNO_3_ and HClO_4_. Subsequently, 6 mL of the mixture of HNO_3_ and HClO_4_ (2,1, *v*/v) was added to digestion tube into the digester block. The resulting solution was allowed to cool and the extract was transferred to a 50 mL volumetric flask to quantification of minerals.

### 2.4 Preparation of extracts and fractionation

To obtain aqueous extracts, fresh fruits were macerated and filtered. For hydroethanolic extracts, approximately 2.0 g of lyophilized fruits were subjected to extraction with ethanol:water (80,20, v/v) for 30 min in an ultrasonic bath (model SSBuc-6 L, MyLabor) and then filtered. After filtration, the residues were re-extracted twice, and the filtrates were combined for subsequent solvent evaporation to obtain the hydroethanolic extract. The aqueous and hydroethanolic extracts were used in antioxidant capacity assays and for the determination of total carotenoid and phenolic compound content.

The hydroethanolic extract was fractionated on a Sephadex LH-20 column (Sigma-Aldrich, Steinheim, Alemanha) using a water-methanol gradient, starting with 100 % water and gradually decreasing the water content by 10 % increments until reaching 100 % methanol. This process resulted in 10 fractions, which were grouped by similarity into five fractions: F1–4 (246.4 mg), F5 (878.8 mg), F6 (63.5 mg), F7 (29.3 mg), and F8–10 (24.0 mg). The fractions were subsequently analyzed by NMR (Nuclear Magnetic Resonance) and HRMS (High Resolution Mass Spectrometer).

### 2.5 Antioxidant capacity (DPPH• and ABTS•^+^)

The antioxidant activity of the samples was evaluated based on its radical scavenging capacity using the DPPH• (1,1-diphenyl-2-picrylhydrazyl) assay (Molyneux, 2004) and the ABTS•^+^ (2,2′-azino-bis(3-ethylbenzothiazoline-6-sulfonic acid) assay (Re et al., 1999). For the DPPH• assay, 100 μL of the sample was added to 3900 μL of a DPPH• solution (100 μM) and incubated in the dark for 30 min. Absorbance was then measured using an ultraviolet-visible (UV–Vis) spectrophotometer (model NI 2200, Nova instruments) at 515 nm. For the ABTS•^+^ assay, 30 μL of the sample was added to 3.0 mL of the radical solution (0.700 Abs) and incubated in the dark for 6 min. Absorbance was then measured using a UV–Vis spectrophotometer (model NI 2200, Nova instruments) at 734 nm. For both assays, calibration curves were generated using Trolox (a standard antioxidant) at concentrations of 100, 500, 1000, 1500, and 2000 μM. Results were expressed as micromoles of Trolox equivalent per gram of sample (μmol TE/g).

### 2.6 Total phenolic contents (TPC)

The quantification of Total Phenolic Compounds (TPC) was performed following the methodology described by Velioglu et al. (1998). A 200 μL aliquot of the sample was added to 1.5 mL of a Folin-Ciocalteu reagent/water mixture (1:10) and incubated in the dark for 5 min. After this period, 1.5 mL of sodium bicarbonate solution (60 g/L) was added, and the mixture was incubated in the dark for 90 min. Absorbance was then measured using an ultraviolet-visible (UV–Vis) spectrophotometer (model NI 2200, Nova instruments) at 725 nm. A standard curve was generated using gallic acid (62.5 to 1000 μg/mL). Results were expressed as milligrams of gallic acid equivalents per gram of sample (mg GAE/g).

### 2.7 Total carotenoids content (TCC)

The quantification of Total Carotenoids Content (TCC) was performed by mixing samples, water, and hexane in a 1:5:6 (*v*/v/v) ratio. The mixture was then vortexed for 1 min to facilitate extraction and centrifuged for 1 min to separate the supernatant. The absorbance of the supernatant was measured using a microplate reader (model SK201, Lleida) at 450 nm. Hexane was used as the blank, and β-carotene was used to construct a calibration curve (3.9 to 62.5 μg/mL) (Carvalho et al., 2020).

### 2.8 Cytotoxicity evaluation

The freeze-dried fruit extracts were subjected to the colorimetric MTT assay (3-(4,5-dimethylthiazol-2-yl)-2,5-diphenyltetrazolium bromide) to determine the cytotoxic concentration that reduces cell viability by 50 % (CC_50_). The assay was performed on African green monkey kidney epithelial cells (Vero cells), human liver cancer cells (HepG2), human embryonic kidney cells (HEK), and murine macrophage cells (RAW 264.7).

Cells were cultured in Dulbecco's Modified Eagle Medium (DMEM) supplemented with 10 % fetal bovine serum (FBS) and 0.05 % DMSO, and maintained at 37 °C in a humidified atmosphere with 5 % CO₂. A suspension of approximately 1 × 10^4^ cells/mL was seeded in 96-well plates (100 μL/well). After 24 h or once reaching confluence, cells were treated with serial dilutions of the freeze-dried fruit extracts, prepared in incomplete DMEM (without FBS), starting at a concentration of 100 μg/mL. The treatment was carried out for 24 h at 37 °C under 5 % CO₂.

Following incubation, the treatment medium was removed, and 100 μL of MTT solution (0.5 mg/mL) was added to each well. The plates were incubated again for 4 h under the same conditions. After incubation, the MTT solution was removed and replaced with 100 μL of DMSO to dissolve the resulting formazan crystals. Plates were gently shaken for 20 min, and absorbance was measured at 540 nm using a spectrophotometer.

CC_50_ values were calculated by non-linear regression analysis of the percentage of cell viability inhibition at varying extract concentrations. Each CC_50_ value represents the mean of three independent experiments (Silva et al., 2023).

Control groups included a negative control (medium containing 0.05 % DMSO, corresponding to 0 % cytotoxicity) and a positive control (medium with 10 % DMSO, resulting in 100 % cytotoxicity).

### 2.9 Volatile analysis by GC–MS

To identify volatile compounds, 100 g of fresh fruits were crushed and subjected to hydrodistillation using a Clevenger apparatus for 3 h. Volatiles were extracted from the hydrolate with 1 mL of hexane GC grade (Sigma-Aldrich, Steinheim, Alemanha).

Separation and identification of volatile compounds were performed using a Gas Chromatography-Mass Spectrometry (GC–MS) system (GC-2030 coupled to GCMS-QP2020 NX, Shimadzu) equipped with an SH-RTx-5Sil MS fused silica capillary column (30 m × 0.25 mm, 0.25 μm film thickness). The inlet and detector temperatures were set at 260 °C. Helium was used as the carrier gas at a flow rate of 1.0 mL/min. The column temperature was programmed to increase from 60 °C to 250 °C at a rate of 3 °C/min. A 1.0 μL aliquot of the hexane extract was injected with a 1:10 split ratio. Ionization energy was set at 70 eV, and the mass scan range was 32–420 Da (Azevedo et al., 2018).

Compound identification was based on their GC retention indices, determined using a homologous series of C_6_–C_30_ *n*-alkanes (Sigma-Aldrich, Steinheim, Germany). The arithmetic index was calculated using Eq. 1 (Viegas & Bassoli, 2007). Compounds exhibiting spectral similarity greater than 90 % with the NIST20 library and differing by less than 10 index units from the calculated arithmetic index (Adams, 2017) were considered to be correctly identified (Teruel-Andreu et al., 2024).

(2) $$\text{Arithmetic index}=100*\frac{t_{c}-t_{n}}{t_{n+1}-t_{n}}+100*n$$

where: ***t***_***c***_ – retention time of the compound of interest (min); ***t***_***n***_ – retention time of the *n*-alkane eluting immediately before the compound of interest (min);***t***_***n+1***_ – retention time of the *n*-alkane eluting immediately after the compound of interest (min); and ***n*** – number of carbon atoms in the *n*-alkane eluting immediately before the compound of interest.

### 2.10 Nuclear magnetic resonance (NMR) analysis

The spectra were acquired using a Bruker Avance III HD Nuclear Magnetic Resonance (NMR) spectrometer (11.74 T, BBFO Plus SmartProbe), observing ^1^H and ^13^C at 500.13 and 125.77 MHz, respectively. To obtain the chemical profiles, fresh fruits were macerated, and 550 μL of the juice was added to a 5 mm tube with 50 μL of D_2_O containing 0.6 mM of TMSP-d4. The fractions of the hydroethanolic extract were solubilized with an appropriate deuterated solvent [F1–4 (D_2_O – Deuterated water) and F5, F6, F7 and F8 (CD_3_OD – Deuterated methanol)]. The TopSpin 4.3.0 software (Bruker BioSpin Inc.) and the Chenomx NMR Suite 10.1 software (Chenomx Inc.) were used for data processing. The experiments conducted were ^1^H, COSY, HSQC and HMBC. Chemical shifts were expressed in parts per million (ppm), using the TMS (Tetramethylsilane) signal at 0.00 ppm as a reference, and coupling constants were indicated in hertz (Hz) (Ramos et al., 2019).

### 2.11 High resolution mass spectrometry (HRMS) analysis

The HRMS analysis was performed on an ESI-MicroTOF-Q II hybrid quadrupole time-of-flight mass spectrometer (Bruker Daltonics Inc.). Sample (1 mg/mL) was diluted in methanol/water (1:1, *v*/v) with 0.1 % CH_2_O_2_ and 3 mM NH_4_HCO_2_. The mass spectrometer parameters were as follows: capillary voltage (−3.5 kV for negative and 4.5 kV for positive ion modes); nebulizer gas (nitrogen, 2.0 bar); dry gas (N_2_, 6.0 L/min) and mass range (*m*/*z* = 100–800 Da) (Mar et al., 2020). The instrument was calibrated with HCOONa. Data acquisition and processing were performed using the software Bruker Compass Data Analysis 4.1.

## 3 Results and discussion

### 3.1 Physicochemical characterization of fruits

The Amazon fig is a small fruit with a longitudinal dimension of 8.6 mm and a weight of 0.54 g. Its physicochemical characteristics are presented in Table 1. The fruit is acidic, exhibiting a lower pH than that of *Ficus carica* fruits, which ranged from 3.87 to 5.13 in the study conducted by Ferraz et al. (2021). The authors reported that the soluble solids content varied from 5.88 to 11.50°Brix.

**Table 1:** Physical, physicochemical and proximate composition of the Amazon fig.

Parameters	Mean ± SD
Mass (g)	0.5 ± 0.1
Dimension (mm)	8.6 ± 0.5
Titratable acidity (%)	0.1 ± 0.0
pH	3.7 ± 0.1
Soluble solids (°Brix)	6.6 ± 0.0
Moisture (g/100 g)	78.65 ± 0.81
Carbohydrates (g/100 g)	19.51 ± 0.16
Protein (g/100 g)	1.25 ± 0.02
Ash (g/100 g)	0.71 ± 0.01
Lipids (g/100 g)	0.32 ± 0.02
Energy (kcal/100 g)	86.02 ± 0.47
**Color**	
L*	7.47 ± 0.05
a*	17.01 ± 0.30
b*	7.52 ± 0.09
C*	18.60 ± 0.31
h*	23.84 ± 0.16

Results are expressed as mean ± standard deviation (*n* = 3).

The proximate composition of the Amazon fig was similar to that of other *Ficus* fruits, particularly due to its high moisture content (78.65 %), comparable to *F. carica* (Palmeira et al., 2019), *F. auriculata* and *F. semicordata* (Puangpradab et al., 2018), which have moisture levels of 69.50 %, 85.01 %, and 76.99 %, respectively. Carbohydrates were the second most abundant component, while lipids, proteins, and ash were present in lower proportions. This fruit has a caloric value of 86.02 kcal/100 g, which is lower than that of *F. carica* (114.1 kcal/100 g) (Palmeira et al., 2019), *F. hispida* (175.44 kcal/100 g) and *F. fistulosa* (142.29 kcal/100 g) (Rusmadi et al., 2020).

The color parameters of the fruit indicated low L* values (lightness), suggesting that it is not very bright. The fruit exhibited positive a* (red) and b* (yellow) values, indicating a tendency toward an orange hue, with a greater contribution from red. This was further supported by the hue angle (h*), which was measured at 23.84°.

### 3.2 Mineral content

The Table 2 presents the mineral content identified in *Ficus subapiculata*. The mineral profile of *Ficus subapiculata* is comparable to that of *Ficus carica* – a commercially cultivated fig – where K and Ca are also the predominant elements (Sedaghat & Rahemi, 2018; Turco et al., 2020). Together, these minerals play essential roles in bone and tooth formation, cellular function, nerve and muscle activity, enzymatic processes, and blood coagulation (Johnson, 2023).

**Table 2:** Mineral content in freeze-dried and fresh Amazon fig.

Mineral	Mean ± SD freeze-dried	Mean ± SD fresh^a^	RDV (Food and Drug Administration, 2022)	%DV in 50 g of dry fruit	%DV in 50 g of fresh fruit
	g/kg	g/kg	g		
K	13.17 ± 0.28	2.81 ± 0.06	4.70	14.01	2.99
Ca	8.50 ± 0.34	1.81 ± 0.07	1.30	32.69	6.98
Mg	1.90 ± 0.09	0.40 ± 0.02	0.42	22.62	4.76
P	0.86 ± 0.05	0.18 ± 0.01	1.25	3.44	0.73
	mg/kg	mg/kg	mg		
Fe	65.90 ± 2.19	14.07 ± 0.47	18.00	18.31	3.91
Mn	43.69 ± 1.43	9.33 ± 0.30	2.30	94.98	20.28
Zn	25.75 ± 1.20	5.50 ± 0.26	11.00	11.70	2.50
Cu	9.40 ± 0.39	2.01 ± 0.08	0.90	52.22	11.15

a Converted from dry material to wet material from moisture content using factor of 0.21. Results are expressed as mean ± standard deviation (n = 3). **RDV**: Recommended Daily Value. **%DV**: Percentage of the Daily Value.

The primary micromineral found in Amazon fig was potassium (K), with concentrations of 13.17 ± 0.28 g/kg in freeze-dried fruits and 2.81 g/kg in fresh fruits. A 50 g portion of these fruits provides 14.01 % and 2.99 % of the daily recommended intake of this essential nutrient, respectively. The K content in Amazon fig is comparable to that of banana pulp – widely recognized as a potassium source – where different varieties contain between 2.95 and 5.10 g/kg (Nadeeshani et al., 2021; Sulaiman et al., 2011). However, its K content is lower than that of *Ficus carica* (8.12 to 3.50 g/kg) (Turco et al., 2020).

Calcium (Ca) and magnesium (Mg) are also noteworthy, as they are the most abundant macrominerals in Amazon fig. A 50 g portion of freeze-dried fruits supplies 32.69 % and 22.62 % of the daily requirement for Ca and Mg, respectively. A recent study revealed that over 5 billion people worldwide do not consume adequate Ca (Lassale & Gaye, 2024), and an estimated 2.2 billion people (30 % of the global population) have insufficient Mg intake (Passarelli et al., 2024). Therefore, identifying alternative dietary sources of these essential minerals is critical, particularly for vulnerable populations with limited access to nutrient-rich foods.

In this study, iron (Fe) and manganese (Mn) were the primary microminerals identified. However, Mn and copper (Cu) contributed the most to daily nutritional needs. A 50 g portion of Amazon fig provides 20.28 % and 11.15 % of the daily requirement for Mn and Cu, respectively, with these percentages increasing significantly in freeze-dried fruits.

Additionally, the findings highlight Amazon fig as a potential dietary source for supplementing mineral intake, which could have various applications in food formulations.

### 3.3 Antioxidant capacity, phenolic content and carotenoids

The results for the antioxidant capacity and total contents of phenolic compounds and carotenoids in the Amazon fig are presented in Table 3. Among the parameters analyzed, only carotenoids were not detected.

**Table 3:** Antioxidant, Carotenoids and Phenolic content of Amazon fig.

Extract	DPPH• (μmol TE/g)	ABTS•^+^ (μmol TE/g)	TPC (mg GAE/g)	TCC (μg/mL)
Aqueous	1061.9 ± 1.6	1433.0 ± 17.6	442.6 ± 0.1	ND
Hydroethanolic	1539.1 ± 5.7	1994.9 ± 15.9	709.5 ± 1.2	ND

Results are expressed as mean ± standard deviation (*n* = 3). **TPC**: Total Phenolic Compounds. **TCC**: Total Carotenoids Content. **ND**: Not Detected. **μ**mol **TE/g**: micromoles of Trolox equivalent per gram of sample. **mg GAE/g**: milligram of Gallic Acid equivalent per gram of sample. DPPH• (Standard curve, y = −0.0006× + 0.9035, R^2^ = 0.9918), ABTS•^+^ (Standard curve, y = −0.0003× + 0.6659, R^2^ = 0.9966), TPC (Standard curve Standard curve, y = 0.0052× + 0.0012, R^2^ = 0.9999).

The highest total phenolic content (TPC) was observed in the hydroethanolic extract (709.5 mg GAE/ g) compared to the aqueous extract (442.6 mg GAE/g). Both extracts exhibited higher TPC than reported for *Ficus fistulosa* fruits (77.66 mg GAE/g) (Puangpradab et al., 2018) and methanolic extracts of *Ficus deltoidea* fruits, which ranged from 70.90 to 299.78 mg GAE/g (Mohd Dom et al., 2020). The higher phenolic content observed in the hydroethanolic extract can be attributed to the intermediate polarity of the 80 % ethanol solution compared to pure water. This solvent mixture presents a polarity level between its individual components, allowing interactions with phenolic compounds across a wide polarity range. As a result, it enables the extraction of both highly polar and moderately or even less polar phenolics (El Mannoubi, 2023). Several studies have demonstrated that this concentration is optimal for phenolic compound extraction from diverse plant matrices (Brahmi et al., 2022; Bucić-Kojić et al., 2011; On-Nom et al., 2024), which explains its traditional use in fig extractions (Bucić-Kojić et al., 2011; Q. Zhang et al., 2024).

Similarly, the antioxidant capacity was superior in the hydroethanolic extract (DPPH• – 1539.1 μmol TE/g; ABTS•^+^ − 1994.9 μmol TE/g) compared to the aqueous extract (DPPH• – 1061.9 μmol TE/g; ABTS•^+^ − 1433.0 μmol TE/g). Both extracts showed higher antioxidant activity than those reported for aqueous infusions of *Ficus palmata* fruits (DPPH – 699.51 μmol TE/g; ABTS – 1059.33 μmol TE/g) (Tewari et al., 2020).

Phenolic content has been positively associated with antioxidant capacity (Y. Zhang et al., 2025), as well as with anti-inflammatory activity (Dongho Dongmo et al., 2025). This is because phenolic compounds help stabilize free radicals in chemical assays by generating neutral species and resonance-stabilized intermediates (Platzer et al., 2021; Sirisha et al., 2010). Antioxidant capacity is a highly valued property due to its potential role in the prevention and/or treatment of cardiovascular diseases, cancer, neurological decline, and diabetes (Wootton-Beard & Ryan, 2011).

These findings suggest that the fruit extracts, particularly the hydroethanolic one, hold strong potential for application in the development of functional foods, where phenolic compounds may serve as bioactive ingredients contributing to health promotion and the prevention of chronic diseases.

### 3.4 Cytotoxicity evaluation

**Fig. 2:**
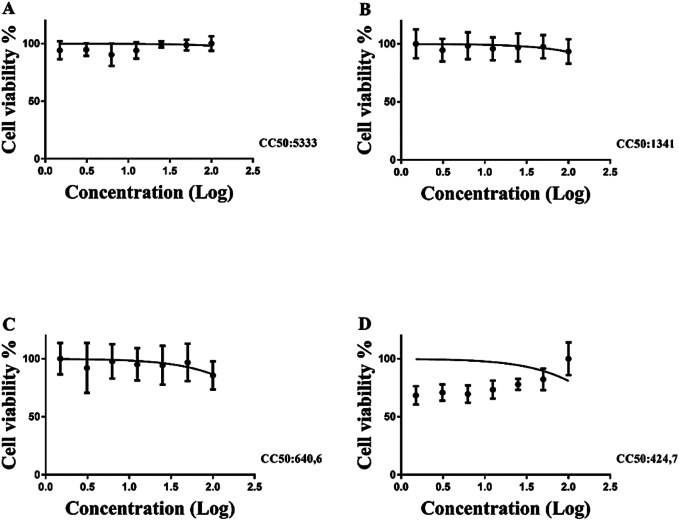
Cytotoxicity screening (CC_50_ in μg/mL). Cell lineage: Vero Cells (**A**), HEPG2 Cell (**B**), HEK Cells (**C**), RAW Cells (**D**). The CC_50_ values corresponds to the average of three independent experiments.

The Fig. 2 presents the cytotoxicity results for freeze-dried *Ficus subapiculata* fruits. The tested cell lines included African green monkey kidney epithelial cells (Vero Cells, A), human liver cancer cells (HEPG2, B), human embryonic kidney cells (HEK, C), and murine macrophage cells (RAW, D). The cytotoxic concentration that decreases cell viability by 50 % (CC₅₀) was determined as 5333 μg/mL, 1341 μg/mL, 640.6 μg/mL, and 424.7 μg/mL for Vero Cells, HEPG2, HEK, and RAW cell lines, respectively.

The results indicate that the freeze-dried fruits exhibited no cytotoxic activity against the tested cells under the given conditions, with CC₅₀ values higher than those reported for other edible samples, such as *Pouteria caimito* pulp (CC₅₀ values were 877.2 μg/mL in Vero cells, 584.4 μg/mL in HepG2 cells, 605.6 μg/mL in HEK cells, and 420.8 μg/mL in RAW cells) (Oliveira et al., 2025).

### 3.5 Analysis by GC/MS

The volatile compounds identified in the Amazon fig by GC–MS are presented in Table 4. A total of 26 compounds were identified, the majority of which (19) belong to the sesquiterpene class (compounds 2 to 20), accounting for 50.08 % of the total area. These are followed by three esters (17.07 % of the area), and one compound each from the alcohol, aldehyde, and sesquiterpenoid classes. In the review by Cruz et al. (2022), the presence of various constituent groups in fig essential oil was reported, including hydrocarbons, aldehydes, alcohols, ethers, esters, ketones, carboxylic acids, phenolic acids, monoterpenes, and sesquiterpenes. However, in general, aldehydes are the main constituents, with few terpenes present, as is the case for conventional fig (*Ficus carica*) (Teruel-Andreu et al., 2024).

**Table 4:** Volatile compounds on Amazon fig.

**N.**	**RT**	**MF**	**Compound**	**Area (%)**	**AI**		**SI**	**Fragments (*m*/*z*)**
					**Exp.**	**Lit.**		
**1**	19.96	C_10_H_16_O	*E,E*-2,4-decadienal	0.37 ± 0.02	1314	1315	91	152, 81, 67, 55, 41
**2**	20.70	C_15_H_24_	*δ*-elemene	0.38 ± 0.03	1331	1335	91	204, 189, 175, 161, 148, 136, 121, 105, 93, 77, 67, 53, 41
**3**	22.39	C_15_H_24_	*α*-copaene	1.04 ± 0.04	1371	1374	94	204, 189, 175, 161, 147, 133, 119, 105, 91, 81, 69, 55, 41
**4**	22.99	C_15_H_24_	*β*-elemene	8.37 ± 0.45	1385	1389	95	204, 189, 175, 161, 147, 133, 121, 107, 93, 81, 67, 53, 41
**5**	23.98	C_15_H_24_	*cis*-α-bergamotene	0.29 ± 0.01	1408	1411	91	204, 189, 161, 133, 119, 107, 93, 79, 69, 55, 41
**6**	24.20	C_15_H_24_	*E*-caryophyllene	2.69 ± 0.07	1414	1417	96	204, 189, 175, 161, 147, 133, 120, 105, 93, 79, 69, 55, 41
**7**	24.82	C_15_H_24_	*trans*-α-bergamotene	2.38 ± 0.06	1429	1432	95	204, 189, 161, 148, 133, 119, 107, 93, 79, 69, 55, 41
**8**	25.67	C_15_H_24_	*E*-*β*-farnesene	2.36 ± 0.04	1449	1454	91	204, 189, 170, 161, 147, 133, 120, 107, 93, 79, 69, 55, 41
**9**	26.43	C_15_H_24_	4α,8-Dimethyl-2-(prop-1-en-2-yl)-1,2,3,4,4a,5,6,7-octahydronaphthalene	1,77 ± 0.08	1468	1473^a^	94	204, 189, 175, 161, 147, 133, 119, 105, 91, 81, 67, 55, 41
**10**	26.71	C_15_H_24_	Germacrene D	1.45 ± 0.01	1475	1484	92	204, 161, 147, 133, 119, 105, 91, 79, 67, 55, 41
**11**	27.03	C_15_H_24_	*β*-selinene	5.36 ± 0.04	1482	1489	96	204, 189, 175, 161, 147, 133, 121, 105, 93, 79, 67, 55, 41
**12**	27.14	C_15_H_24_	Viridiflorene	0.35 ± 0.01	1486	1496	91	204, 189, 175, 161, 147, 135, 119, 107, 93, 79, 67, 55, 41
**13**	27.33	C_15_H_24_	*α*-selinene	5.08 ± 0.28	1490	1498	94	204, 189, 175, 161, 147, 133, 121, 107, 93, 81, 67, 55, 41
**14**	27.47	C_15_H_24_	*α*-muurolene	0.96 ± 0.09	1493	1500	95	204, 189, 161, 147, 133, 119, 105, 91, 81, 67, 55, 41
**15**	27.88	C_15_H_24_	*β*-bisabolene	2.21 ± 0.09	1503	1505	95	204, 189, 175, 161, 147, 134, 119, 107, 93, 79, 69, 53, 41
**16**	28.02	C_15_H_24_	*γ*-cadinene	2.72 ± 0.10	1507	1513	95	204, 189, 176, 161, 148, 133, 119, 105, 91, 79, 67, 55, 41
**17**	28.26	C_15_H_24_	*δ*-cadinene	1.92 ± 0.07	1513	1522	92	204, 189, 176, 161, 145, 134, 119, 105, 91, 81, 69, 55, 41
**18**	28.62	C_15_H_24_	*E*-*γ*-bisabolene	1.28 ± 0.09	1522	1529	92	204, 189, 175, 161, 148, 135, 119, 107, 93, 79, 69, 55, 41
**19**	32.62	C_15_H_26_O	*γ*-eudesmol	6.81 ± 0.29	1625	1630	96	222, 204, 189, 175, 161, 147, 133, 119, 105, 91, 81, 67, 59, 43
**20**	33.48	C_15_H_26_O	*α*-eudesmol	2.66 ± 0.09	1648	1652	91	222, 204, 189, 175, 161, 149, 133, 122, 107, 93, 81, 67, 59, 43
**21**	33.57	C_15_H_26_O	Neointermedeol	1.05 ± 0.01	1650	1658	91	222, 204, 189, 175, 161, 147, 135, 121, 109, 93, 81, 71, 55, 43
**22**	41.62	C_16_H_34_O	1-hexadecanol	0.97 ± 0.05	1876	1874	95	224, 196, 168, 154, 139, 125, 111, 97, 83, 69, 55, 43
**23**	43.13	C_17_H_34_O_2_	Methyl palmitate	0.60 ± 0.07	1921	1921	91	270, 239, 227, 199, 185, 171, 157, 143, 129, 101, 87, 74, 55, 43
**24**	44.39	C_16_H_32_O_2_	Hexadecanoic acid	4.00 ± 0.18	1960	1959	95	256, 227, 213, 185, 171, 157, 143, 129, 115, 97, 83, 73, 55, 41
**25**	48.36	C_19_H_34_O_2_	Methyl linoleate	6.76 ± 0.16	2086	2095	94	294, 263, 220, 178, 164, 150, 135, 123, 109, 95, 81, 67, 55, 41
**26**	50.40	C_20_H_36_O_2_	Linoleic acid, ethyl ester	5,71 ± 0.11	2154	2159^a^	96	307, 263, 220, 178, 164, 150, 135, 123, 109, 95, 81, 67, 55, 41

**MF**: Molecular Formula. **AI**: Arithmetic Index. **SI**: Similarity Index. **m/z**: Mass-to-charge ratio. **Exp.**: Experimental Value. **Lit.**: Adams, 2017. ^a^https://pubchem.ncbi.nlm.nih.gov/.

In Amazon fig essential oil, sesquiterpenes represent the largest group of secondary metabolites and are associated with plant defense mechanisms due to their antimicrobial activity. Recent studies have highlighted the potential health benefits of sesquiterpenes and their derivatives, particularly in mitigating metabolic syndromes, including cardiovascular complications, neurological disorders, diabetes, and cancer (Abu-Izneid et al., 2020).

Among the sesquiterpenes found in the Amazon fig, β-elemene (8.37 %) was the most abundant, a compound previously identified in *Ficus carica* (Nawade et al., 2020). *In vitro* and *in vivo* studies, along with molecular analyses, have demonstrated its potential in modulating inflammatory responses (Fang et al., 2018; Shao et al., 2021; Zhou et al., 2021) and in cancer treatment (Zhai et al., 2019). β-Elemene is a valuable target for technological development and warrants further investigation for its potential applications in the food and pharmaceutical industries, particularly as a natural flavoring agent with potential additional health benefits.

### 3.6 Chemical characterization by NMR and HRMS

**Fig. 3:**
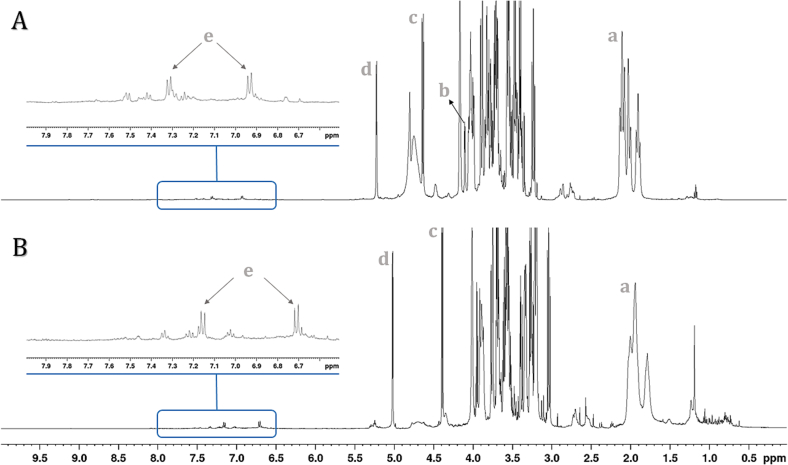
^1^H NMR spectra from 0.00 to 10.00 ppm of the aqueous extract in D_2_O/H_2_O (**A**) and hydroethanolic extract in CD_3_OD (**B**), referenced with TMS (500 MHz, 11.74 T). **a**: quinic acid. **b**: fructose. **c**: β-glucose. **d**:-glucose. **e**: 4-hydroxybenzoic acid.

The ^1^H NMR spectra of the aqueous and hydroethanolic extracts of Amazon fig exhibit similar profiles (Fig. 3), displaying characteristic signals corresponding to carbohydrates, organic acids, and aromatic compounds. In the spectrum (Fig. 3), certain signals stand out due to their intensity, representing the major compounds in the sample.

In the aqueous extract (Fig. 3A), signals commonly found in fruit pulps are observed. The signals between δ_H_ 1.91 and 2.12 ppm (**a**) are characteristic of quinic acid (Tiwari et al., 2021). The signals at δ_H_ 4.10 ppm (d, *J* = 3.5 Hz) (**b**), δ_H_ 4.63 ppm (d, *J* = 8.0 Hz) (**c**), and δ_H_ 5.22 ppm (d, *J* = 3.6 Hz) (**d**) were assigned to fructose, β-glucose, and α-glucose, respectively (Brown et al., 2018; Navarro et al., 2020).

These signals are also present in the hydroethanolic extract (Fig. 3B), albeit with a slightly lower chemical shift due to solvent effects. The signals in the hydroethanolic extract that correspond to those previously observed in the aqueous extract are labeled with the same letter (Fig. 3).

Additionally, less intense signals can be observed in the aqueous extract (Fig. 3A), such as the doublets at δ_H_ 2.93 and δ_H_ 2.79 (*J* = 15.8 Hz), attributed to the hydrogens of citric acid (Campo et al., 2006), as well as the signals at δ_H_ 2.74, δ_H_ 2.78, and δ_H_ 4.34, corresponding to malic acid (Campo et al., 2006), and the multiplet at δ_H_ 6.76, associated with shikimic acid (Wu et al., 2012). In the aromatic region, low-intensity signals are also detected, particularly at δ_H_ 7.32 (d, *J* = 8.5 Hz) and δ_H_ 6.93 (d, *J* = 8.5 Hz) (e), which correspond to the 1,4-disubstituted benzene nucleus of p-hydroxybenzoic acid (Wang et al., 2023).

The high intensity of the signals corresponding to the major compounds, particularly sugars, hinders the detection of secondary metabolites present in Amazon fig. To overcome this limitation, a fractionation process was performed on the hydroethanolic extract to facilitate the identification of additional constituents. The compounds identified in the fractions by NMR and HRMS are listed in Table 5.

**Table 5:** Compounds identified from *Ficus subapiculata* fruit.

Compound Chemical class	Extract fraction (Solvent)	NMR Data					HRMS Data
		Position	δ_H_ in ppm (Multiplicity, coupling in Hz)	COSY	HSQC (δ_C_ in ppm)	HMBC (δ_C_ in ppm)	[M–H]^−^, MF and Error in ppm
**α-d-Glucose** Carbohydrate	F1–4 (D_2_O)	1	5.22 (d, 3.7)	–	–	–	–
		2	3.54 (dd, 3.8 e 9.5)	–	–	–	
		6	3.84 (dd, 2.2, 11.5)	–	–	–	
**β-d-Glucose** Carbohydrate	F1–4 (D_2_O)	1	4.63 (d, 7.9)	–	–	–	–
		2	3.23 (dd, 8.0, 9.5)	–	–	–	
		6	3.91 (dd, 2.3, 12.0)	–	–	–	
**Fructose** Carbohydrate	F1–4 (D_2_O)	3	4.10 (d, 3.6)	–	–	–	–
		4	4.01 (m)	–	–	–	
***p*-hydroxybenzoic acid** *Phenolic acid*	F1–4 (D_2_O)	2 and 6	7.23 (dd, 8.6, 1.4)	–	–	–	–
		3 and 5	6.85 (dd, 8.6, 1.6)	–	–	–	
**Malic acid** *Organic acid*	F1–4 (D_2_O) and F5 (CD_3_OD)	2	4.49 (dd, 7.0, 4.5)	–	–	–	133.0142, C_4_H_5_O_5_, −0.3
		3a	2.75 (dd, 16.5, 7.0)	–	–	–	
		3b	2.83 (dd, 16.5, 4.5)	–	–	–	
**Quinic acid** *Organic acid*	F1–4 (D_2_O) and F5 (CD_3_OD)	2 and 6	1.85–2.15 (m)	–	–	–	191.0561, C_7_H_11_O_6_, −4.8
		4	3.58–3.54 (m)	–	–	–	
		3 and 5	4.16 (m)	–	–	–	
**Citric acid** *Organic acid*	F5 (CD_3_OD)	3a	2.93 (d, 15.8)	–	–	–	–
		3b	2.75 (d, 15.8)	–	–	–	
**Shikimic acid** *Organic acid*	F5 (CD_3_OD)	2	6.76 (m)	–	–	–	–
		3	4.38 (m)	–	–	–	
		5	3.83 (m)	–	–	–	
**Protocatechuic acid** *Phenolic acid*	F5 (CD_3_OD)	–	–	–	–	–	153.0200, C_7_H_5_O_4_, −4.3
**Vanillic acid** *Phenolic acid*	F5 (CD_3_OD)	–	–	–	–	–	167.0350, C_8_H_7_O_4_, 0.2
**3-*O*-methylkaempferol** *Flavonol derivative*	F6 (CD_3_OD)	6	6.27 (d, 2.0)	6.48	98.8	93.6 (8), 104.1 (10), 161.5 (5)	299.0549, C_16_H_11_O_6_, 4.2
		8	6.48 (d, 2.0)	6.27	93.6	98.9 (6), 157.1 (9) 175.1 (4)	
		2′, 6’	8.00 (d, 8.7)	6.85	131.7	114.6 (3′), 149.1 (2), 161,9 (4′)	
		3′, 5’	6.88 (d, 8.7)	7.88	114.6	131.7 (6′), 122.1 (1′), 149.0 (2)	
		1”	3.91 (s, OCH_3_)	–	55.3	147.4 (3)	
**Quercetin** *Flavonol*	F6 (CD_3_OD)	6	6.29 (d, 2.0)	6.32	98.9	176.1 (4), 89.9 (9), 105.8 (10)	301.0343, C_15_H_9_O_7_, 3.6
		8	6.51 (d, 2.0)	6.18	89.9	176.1 (4), 167.4 (7), 105.8 (10)	
		2’	7.55 (d, 2.1)	7,44	116.5	120.6 (1′), 150.9 (4′)	
		5’	6.85 (d, 8.8)	7,44	114,7	123.2 (6′), 144.6 (3′)	
		6’	7.44 (dd, 8.8, 2.1)	6.85, 7.55	123.2	150.0 (2), 116.5 (2′)	
**Rutin** *Flavonol derivative*	F6 (CD_3_OD)	–	–	–	–	–	609.1439, C_27_H_29_O_16_, −3.7
**3-*O*-methylgallic acid** *Phenolic acid*	F7 (CD_3_OD)	–	–	–	–	–	183.0307, C_8_H_7_O_5_, 4.4
**Chlorogenic acid** *Phenolic acid*	F7 (CD_3_OD)	2a	1.97 (m)	1.86, 4.23	41.7	42.9 (6), 72.6 (3), 78.8 (1), 180.0 (7)	353.0874, C_16_H_17_O_9_, 1.2
		2b	1.86 (m)	1.97, 4.23	41.7	42.9 (6), 72.6 (3), 78.8 (1), 180.0 (7)	
		3	4.23 (m)	1.97, 1.86, 3.51	72.6	–	
		4	3.51 (dd, 7.5, 3.0)	4.23, 4.04	71.6	42.6 (6), 41.7 (2)	
		5	4.04 (m)	3.51, 1.96, 1.84	73.2	165.4 (9′), 72.6 (3), 78.8 (1), 41.7 (2)	
		6a	1.96 (m)	4.04, 1.84	42.9	41.7 (2), 71.6 (4), 78.8 (1), 180.0 (7)	
		6b	1.84 (m)	4.04, 1.96	42.9	41.7 (2), 71.6 (4), 78.8 (1), 180.0 (7)	
		2’	7.04 (d, 2.1)	6.97	117.8	–	
		5’	6.76 (d, 8.2)	6.97	118.5	–	
		6’	6.93 (dd, 2.1, 8.2)	6.76, 7.07	125.7	–	
		7’	7.55 (d, 16.0)	6.28	118.7	–	
		8’	6.28 (d, 16.0)	7.55	123.1	–	
**4-*O*-feruloyl-quinic acid** *Phenolic acid*	F7 (CD_3_OD)	2a	1.97 (m)	1.86, 4.23	41.7	42.9 (6), 72.6 (3), 78.8 (1), 180.0 (7)	367.1014, C_17_H_19_O_9_, 5.6
		2b	1.86 (m)	1.97, 4.23	41.7	42.9 (6), 72.6 (3), 78.8 (1), 180.0 (7)	
		3	4.23 (m)	1.97, 1.86, 3.51	72.6	–	
		4	3.51 (dd, 7.5, 3.0)	4.23, 4.04	71.6	42.6 (6), 41.7 (2)	
		5	4.04 (m)	3.51, 1.96, 1.84	73.2	166.3 (9′), 72.6 (3), 78.8 (1), 41.7 (2)	
		6a	1.96 (m)	4.04, 1.84	42.9	41.7 (2), 71.6 (4), 78.8 (1), 180.0(7)	
		6b	1.84 (m)	4.04, 1.96	42.9	41.7 (2), 71.6 (4), 78.8 (1), 180.0 (7)	
		2’	7.16 (d, 2.2)	7.07	117.5	–	
		5’	6.83 (d, 8.8)	7.07	118.3	–	
		6’	7.07 (dd, 2.2, 8.8)	6.83, 7.16	125.8	–	
		7’	7.55 (d, 15.8)	6.34	119.2	–	
		8’	6.34 (d, 15.8)	7.55	122.1	–	
		1”	3.87 (s, OCH_3_)	–	58.0	149.9 (3′)	
**Protocatechuic acid *O*-glucoside** *Phenolic acid*	F7 (CD_3_OD)	2	7.55 (d, 3.1)	7.02	121.5	170.8 (7), 125.8 (6), 152.0 (4)	315.0695, C_13_H_15_O_9_, 8.5
		5	6.70 (d, 8.8)	7.02	119.6	122.0 (1), 146.7 (3)	
		6	7.02 (dd, 8.8, 3.1)	7.55, 6.70	125.9	121.5 (2), 152.0 (4)	
		1’	4.71 (d, 7.4)	–	106.4	152.0 (4)	
**Isoorientin** *Flavone C-glucoside*	F6-F7 (CD_3_OD)	–	–	–	–	–	447.0917, C_21_H_19_O_11_, 3.6

**δ**_**H**_ – ^1^H chemical shift in ppm; **δ**_**C**_ – ^13^C chemical shift in ppm; **MF** – Molecular formula; multiplicity: **s** – simplet, **d** – duplet, **dd** – double duplet, **m** – multiplet.

NMR and HRMS analyses of the fractions identified a total of 18 compounds, including 3 carbohydrates (α-glucose, β-glucose, and fructose), 4 organic acids (malic, quinic, citric, and shikimic acids), 7 phenolic acids (4-hydroxybenzoic, protocatechuic, vanillic, 3-*O*-methylgallic, chlorogenic, 4-*O*-feruloylquinic, and protocatechuic-*O*-glucoside acids), and 4 flavonoids (3-*O*-methylkaempferol, quercetin, rutin, and isoorientin). Most of the identified phenolic compounds have been previously reported in fruits of the *Ficus* genus (Cruz et al., 2022).

The sugars and organic acids identified are essential for maintaining efficient metabolic processes, promoting digestive health, and providing antioxidant protection, thereby contributing to overall well-being and the prevention of chronic diseases (Alam et al., 2022; Shi et al., 2022). Furthermore, the intake of phenolic compounds plays a crucial role in human health, primarily due to their antioxidant potential. Recent studies have shown that phenolic acids and flavonoids possess anticancer properties (Silva et al., 2025), support improvements in cardiometabolic parameters (Laveriano-Santos et al., 2024), exhibit anti-inflammatory and neuroprotective effects, delay aging, and promote healthy longevity by facilitating the clearance of senescent cells (Fan et al., 2022).

## 4 Conclusion

This study provides the first comprehensive chemical characterization of the Amazon fig. The results indicate that this underexplored fig is a promising functional food, serving as a source of essential minerals – particularly Mn, Cu, Ca, and Mg – as well as antioxidant compounds. Given the growing demand for novel food sources with high nutritional and functional value, incorporating Amazon fig into the human diet represents a valuable opportunity to diversify food sources and promote the sustainable use of Amazonian biodiversity. Among the limitations of this study is the fact that both the cytotoxicity and antioxidant potential assessments are preliminary screenings and require further validation through *in vivo* assays. Additionally, several gaps remain to be addressed in future studies, such as the quantification of identified compounds using qNMR-based approaches and/or chromatographic techniques (particularly flavonoids and phenolic acids), the evaluation of their bioavailability in *in vivo* models, and an assessment of their potential and safety for use in food formulations.

## CRediT authorship contribution statement

**Josias Martins dos Anjos Cruz:** Writing – review & editing, Writing – original draft, Methodology, Formal analysis, Data curation, Conceptualization. **Renilto Frota Corrêa:** Writing – review & editing, Methodology. **Valdely Ferreira Kinupp:** Writing – review & editing, Methodology. **Michele Fernandes Pereira:** Methodology, Formal analysis, Data curation. **Kidney de Oliveira Gomes Neves:** Methodology, Formal analysis, Data curation. **Jojo Rodrigues:** Methodology, Data curation. **Bianca Muniz Lacerda Ventura:** Methodology, Data curation. **Tiago Antônio de Oliveira Mendes:** Methodology, Data curation. **Edgar Aparecido Sanches:** Writing – review & editing, Writing – original draft. **Pedro Henrique Campelo:** Writing – review & editing, Writing – original draft. **Jaqueline de Araújo Bezerra:** Writing – review & editing, Writing – original draft, Supervision, Project administration, Methodology, Conceptualization.

## Declaration of competing interest

The authors declare that they have no known competing financial interests or personal relationships that could have appeared to influence the work reported in this paper.

## Data Availability

Data will be made available on request.
